# “Tone‐Arm” Neuromodulation MEMS Microcoil for In Vivo Imaging

**DOI:** 10.1002/smll.202514159

**Published:** 2026-02-15

**Authors:** Xiyuan Liu, Bingdong Chang, Kayeon Kim, Gwendoline A. E. Anand, Su Genelioglu, Changsi Cai, Anpan Han

**Affiliations:** ^1^ Department of Civil and Mechanical Engineering Technical University of Denmark Lyngby Denmark; ^2^ Department of Neuroscience Faculty of Health and Medical Science University of Copenhagen Copenhagen Denmark

**Keywords:** BioMEMS, MEMS micro‐coils, neural inhibition, neural interfaces, two‐photon imaging

## Abstract

Implantable neural interfaces have revolutionized neuromodulation by enabling bidirectional communication between neural circuits and external devices. Yet, existing modalities face important challenges in precision, high invasiveness, and compatibility with advanced in vivo imaging systems. We report a MEMS‐based “tone‐arm” micro‐coil design optimized for vertical cortical insertion, featuring an 800‐µm cantilever with a small cross‐section that reconciles mechanical robustness with two‐photon microscopy compatible miniaturization. Numerical modeling spanning electromagnetic field gradients, thermodynamic safety margins, and buckling resistance guided our MEMS device design process. Our toxic‐etchants‐free 3‐stage and 4‐mask fabrication process yielded micro‐coil devices compatible with in vivo two‐photon imaging. Our vertically integrated probe design overcomes longstanding optical obstruction challenges, preserving >95% imaging field visibility during in vivo studies. This platform enables simultaneous micromagnetic neuromodulation and subcellular‐resolution calcium imaging, opening unprecedented opportunities to dissect neural circuit dynamics during targeted intervention. Thermodynamic modelling showed the record low probe resistance (2 Ω) is safe for implantation and limits tissue heating to < 0.2°C. The low‐invasive cross‐section (70 × 86 µm^2^), and mechanical robust brain insertions were critical to achieving robust neuromodulation, including neuro‐inhibition—a capability long sought for the potential treatment of hyperactivity‐driven neurological disorders.

## 1 Introduction

Implantable neural interface (INI) is an innovative technology that enables bidirectional signaling pathway between nerve tissue and exosomatic computational platforms [1, 2, 3, 4, 5]. INIs offer potential for restoring lost sensory and motor functions [6], providing patients with ways to cybernetic limb actuation [7, 8, 9, 10], communicate [11, 12, 13, 14, 15, 16], or interact with computers using only their thoughts [17, 18, 19, 20]. A major application of INIs is neuromodulation of the central nervous system (CNS), which involves precisely stimulating specific brain regions to alter neural activity. CNS INIs are employed in managing neuropathological conditions through targeted neural circuit modulation, including depression [21], epilepsy [22, 23, 24, 25], stroke [26], chronic pain and motor dysfunction [7, 27, 28, 29, 30, 31].

Electrical stimulation is the foundational and commonly used method of CNS neuromodulation, both in pre‐clinical studies and clinical settings. It involves implanting electrodes near target neural regions to activate surrounding neurons through electric currents. Recent studies focus on durable and flexible INIs [1, 32, 33, 34, 35].

Three emerging neuromodulation paradigms are prevalen [36]. (i) Optogenetic stimulation enables selective neural modulation by introducing light‐sensitive proteins into specific neuron types, allowing precise targeting [37, 38, 39, 40, 41]. (ii) Infrared neural modulation (INM) does not require genetic modification. It uses pulsed infrared light to create localized temperature changes in neural tissue, causing depolarization without direct contact [42, 43, 44, 45]. (iii) Complementing the above, magnetic neuromodulation activates neurons by inducing a dynamic electrical field through Faraday's Law of electromagnetic induction [46, 47, 48, 49]. Implantable MEMS micro‐coils (MMC) for magnetic neuromodulation offer unique advantages by eliminating direct electrical contact with neural tissue, reducing the risk of inflammation from implantation [50, 51, 52, 53]. Additionally, since magnetic fields easily penetrate biological tissues, glial scarring around implants has minimal impact on target tissue activation. Furthermore MMC exhibit a distinct advantage in achieving micrometer‐scale spatial resolution, enabling customization of an MMC architecture to enhance neuromodulation efficacy or improve localized activation in biological tissues [54, 55].

While electrical INIs are efficient for neuroactivation, direct neural inhibition is evidently impossible [38, 56]. Neural inhibition is a key aspect of natural neuromodulation, balancing with neural excitation to regulate brain activity. Neural excitation increases the firing rate of neurons and enhance brain activity [57, 58]; however, excessive neuroexcitation is a hallmark of certain brain dysfunction and disorders such as epilepsy and chronic pain. Transcranial magnetic stimulation is a non‐invasive method used clinically for the treatment of depressions, obsessive‐compulsive disorder, and migraine headaches by neuroinhibition [7, 59, 60, 61, 62, 63]. Preclinical optogenetic neuroinhibition, INM are neural inhibition INIs that suppress overactive neurons, stabilizing brain function and restoring normalcy. These neuroinhibition INI technologies can potentially treat important neurological disorders, such as epilepsy, chronic pain, and schizophrenia, by stabilizing hyperactive brain areas [27, 28, 64, 65].

In our recent study [66, 67], we pioneer the combination of MMC with two‐photon excitation fluorescence microscopy (TPM) to explore their role in neural modulation within the mouse visual cortex in vivo [68]. The high‐resolution imaging capability of TPM and the precise magnetic stimulation provided by the MMC allow us to observe the effects of neural modulation in real‐time, further assessing the potential of MMC enhancing spatial modulation accuracy. Using this unique experimental set‐up, we reported that MMC is able to reliably inhibit natural neural activity, locally neutralize neural activity by electrical stimulation, and suppress seizures in mice brain. Aside from our previous in vivo report [68], which primarily focused on cellular‐level findings and neurophysiological responses, the present study emphaisizes the technological and mechanical advancements of the MMC probe, highlighting the substantial improvement in mechanical properties, which is radically different in design and toxic‐etchant‐free fabrication compared to past works [67, 69, 70], demonstrating enhanced robustness, and stability of the probe under physiological conditions. This study extends beyond the cellular findings presented in our prior work by providing a comprehensive evaluation of the probe's mechanical performance and design innovation. It demonstrates how the new MMC platform integrates safer fabrication methods, establishing a robust foundation for future in vivo and translational applications.

However, these methods have short‐comings. E.g. optogenetics requires gene therapy, and its clinical applications face many challenges [45].

## 2 Material and Methods

### 2.1 Numerical Simulations

To guide our MMC design, we conducted numerical simulations using COMSOL. These simulations calculated the electrical field strength, temperature, and mechanical stress distributions to ensure the device's efficacy, thermal safety, and structural stability during implantation. The aluminum micro‐coil with ‘V’ shaped tips were modelled. The dimensions are given in Figure 2 and Table 1. The entire probe was coated with alumina (100‐nm‐thick) for electrical insulation, and Parylene C (6‐µm‐thick) for biocompatibility and protecting the aluminum metal trace from corrosive physiological fluids [71].

**TABLE 1:** Probe dimensions.

	Length (µm)	Thickness (µm)	Max width (µm)	Min width (µm)
Silicon base	8518	350	3637	800
Silicon arm	6000	350	800	550
Silicon cantilever	806	70	86	<1
Aluminum metal trace	795	1.8	15	15

Various material properties were needed for numerical simulations, and the preset values are presented in Table 2, including specified heat capacity C_p_, thermal conductivity k, electric conductivity σ, dielectric constant ε, density ρ, and Young's modulus E. For standard materials like aluminum, silicon, and Al_2_O_3_, the material properties were directly chosen from the material database in COMSOL Multiphysics software. For Parylene C, the material properties are publicly available from the website of manufacturers like Advanced Coating and Specialty Coating Systems. For brain tissue, we used material properties as reported from literature, and references are given accordingly.

**TABLE 2:** Material properties used for heat transfer simulations.

Material	Cp (J kg^−1^ K^−1^)	k (W m^−1^ K^−1^)	σ (S m^−1^)	ε	Density (kg m^−3^)
Aluminum	900	238	3.774 × 107	5000	2700
Silicon	700	130	1 × 10–4	11.7	2329
Al_2_O_3_	730	35	1 × 10–17	5.7	3965
Parylene C	0.17 [72]	0.082 [73]	1 × 10–14	3.2	1289
Brain tissue	3630 [74]	0.45 [75]	0.25	200 [76]	1081 [77]

### 2.2 Fabrication Processes of Micro‐Coils

**FIGURE 1:**
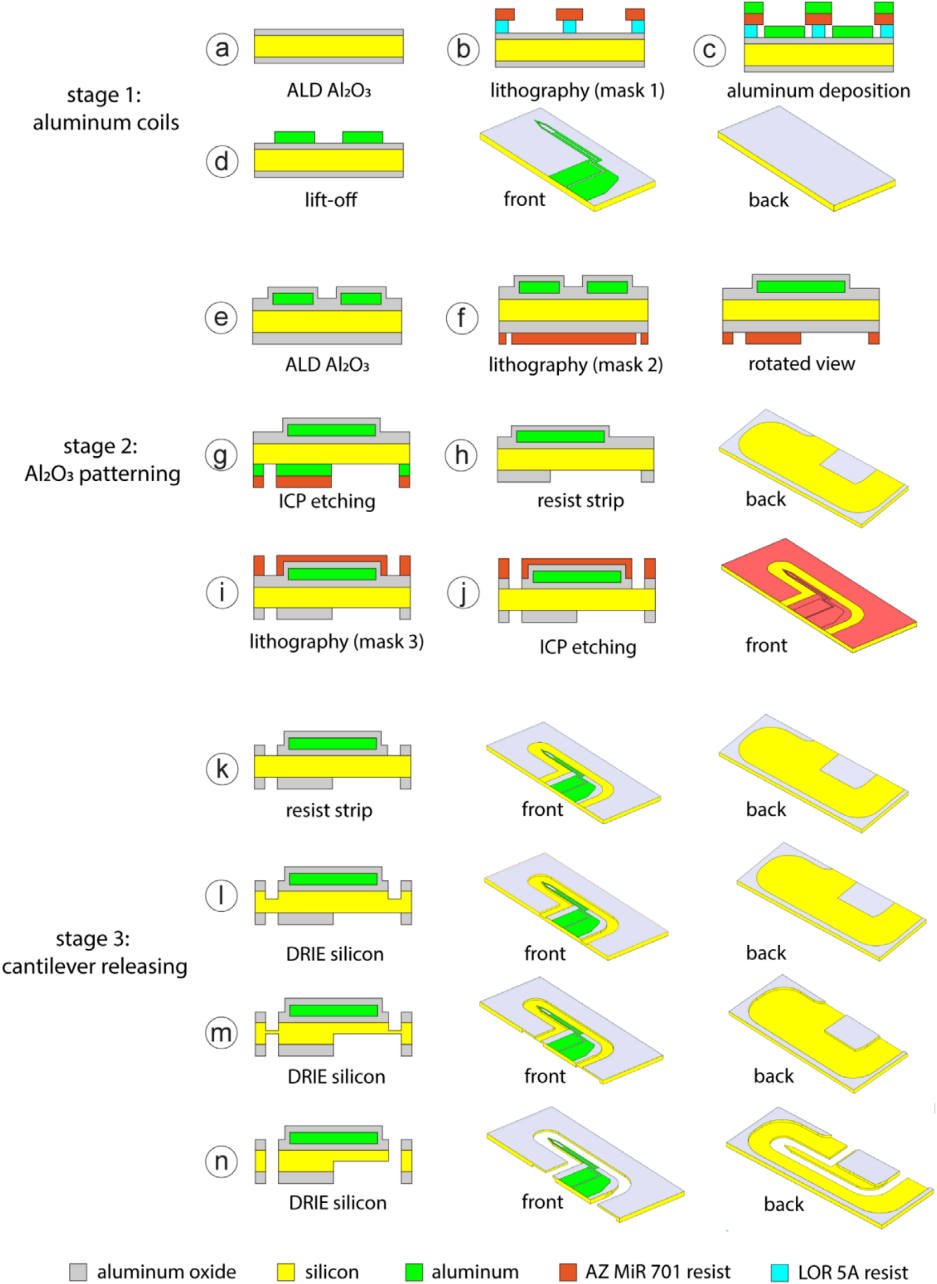
Process flow illustration and probe geometry. (a–d) Stage 1: Aluminum (Al) coil wire patterning. (e–j) Stage 2: Insulating Al_2_O_3_ layer patterning. (k–n) Stage 3: Cantilever release supporting the coil wires.

Figure 1 delineates the 19‐step fabrication sequence employing three photolithographic masks. This innovative approach introduces significant design and process modifications compared to previous studies [12, 70]. Processing initiated on double‐side polished (DSP) silicon wafers with 100 mm diameter and 350 µm thickness.

Initially, atomic layer deposition (ALD) deposition was used to coat 100 nm thick aluminum oxide on both wafer surfaces.This coating step, performed at 300°C in a Picosun R200 thermal ALD reactor (Finland), employed trimethylaluminum (Strem Chemicals, USA) as the source material. Following this, the aluminum micro‐coil structure was defined through the process flow of lithography, metal deposition, and lift‐off (steps b–d). To start with, LOR 30C (Microresist Tech., Germany) was spin‐coated onto the silicon wafers. This process was followed by soft baking and subsequent application of AZ MiR 701 (Microchemicals, Germany) to form a double‐resist stack. The resist stack underwent exposure on a mask‐less aligner (MLA150, Heidelberg Instruments, Germany) and development in AZ 726 MIF developer. Subsequently, physical vapor deposition (PVD) was used to deposit a 3‐ µm‐thick aluminum thin film, followed by a lift‐off process in an ultrasonic bath using N‐Methyl‐2‐pyrrolidone. To ensure efficient lift‐off, the LOR film thickness was maintained at 1.2–1.3 times that of the deposited metal layer.

Next, the Al_2_O_3_ layer was patterned through a five‐stage process. An additional 100‐nm layer of alumina was grown via ALD onto the silicon substrate to encapsulate the aluminum wires (step e). Subsequent steps (f–k) involved defining the pattern on the front side using photolithography and plasma etching. To achieve the required 3 µm margin precision, a 4‐µm‐thick photoresist was Patterning of this alumina film followed, employing a BCl_3_/Ar plasma etch, which exposed both the aluminum wire bonding pads and the underlying silicon on the backside (step j) [78].

Subsequent processing released the silicon cantilevers supporting the coil wires via deep reactive ion etching (DRIE) [49, 79, 80, 81]. Surrounding material was removed to create a 70 µm height difference between probes and substrate (step l). The backside subsequently underwent thinning to 20 µm using the same technique (step m). Finally, the wafer was mounted onto a custom aluminum carrier to mitigate vacuum effects, followed by removal of residual silicon via DRIE. This produced multiple 70‐µm‐thick cantilevers anchored to a silicon support frame (step n). Mirroring the detachment of atomic force microscopyprobes, the micro‐coils were mechanically released from this frame for packaging.

Packaging involves four sequential operations. Initially, the probes are affixed to a custom printed circuit board (PCB) with gold‐plated bonding pads. Subsequently, gold wire ball bonding is performed to establish electrical connections. Following bonding, the wires are encapsulated using epoxy resin. Finally, a commercial service applies a conformal 6‐µm Parylene C coating over the entire assembled device.

During in vivo TPM experiments, the immobilization of the mouse was secured through the use of a custom‐made metal support bar, that was laser‐enlarged to prevent interference with the PCB (Figure 6b). Insertion of the probe into the brain proceeded through the central aperture of the metal headbar.

This modification enabled smooth insertion of the probe into the target brain region.

### 2.3 Animal Preparation and Stimulation Parameters

We used C57BL/6 mice that were injected with Adeno‐associated viral vector (AAV), GCaMP8f (pGP‐AAV‐syn0jGCAMP8f‐WPRE; Addgene #162376‐AAV9), carrying the neuronal‐specific calcium indicator two weeks prior to the recordings. We targeted visual cortex (+1 mm AP, +2 mm ML relative to lambda) with three different depths, 200 nL for each depth.

**FIGURE 2:**
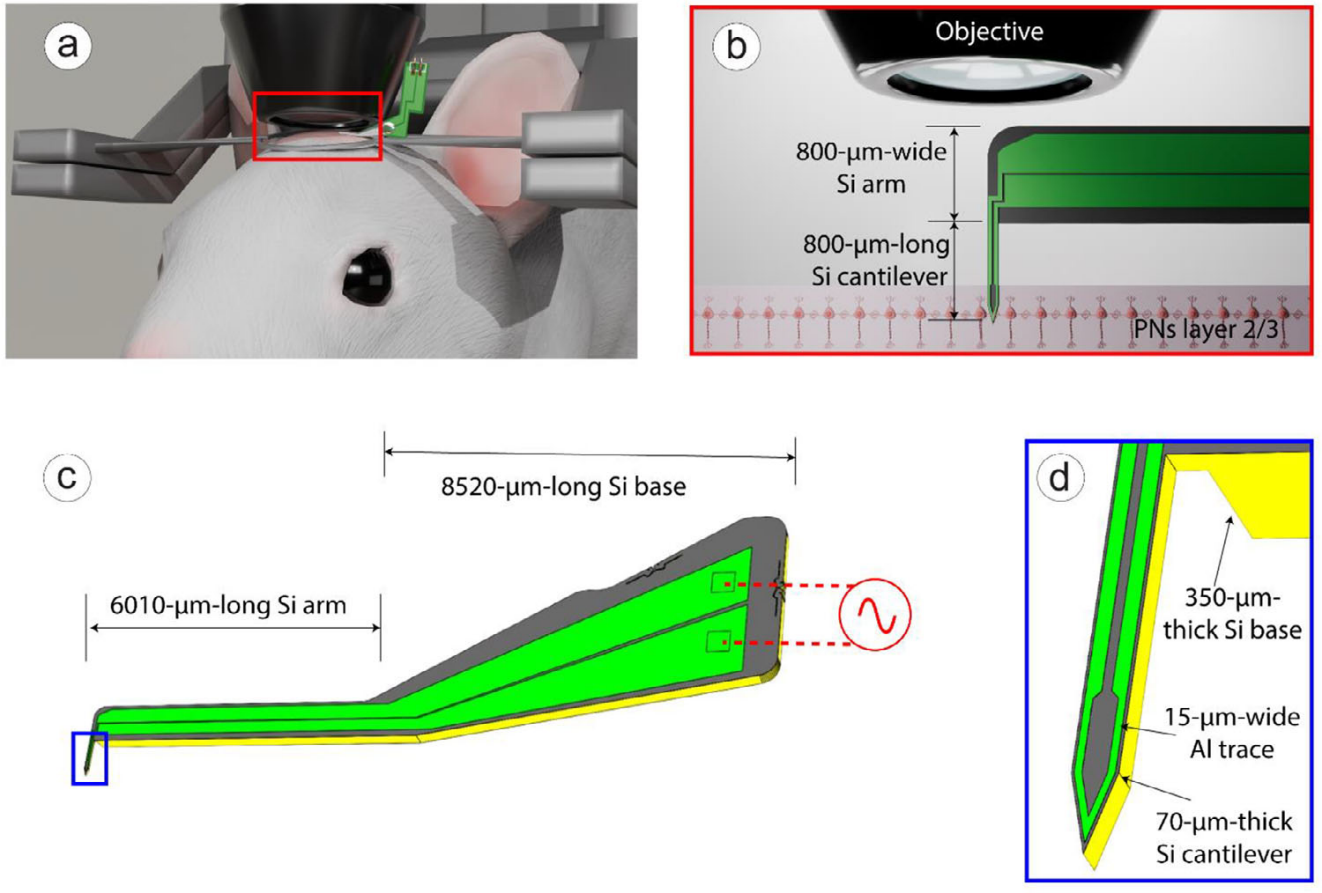
Illustration of Micro‐Coil Probes. (a) Schematic view of the experimental set‐up. (b) Schematic design of the tonearm‐like MMC vertically inserted into pyramidal neurons (PNs) layer 2 to 3. (c) Illustration of the tonearm‐like MMC, and the AC signal input through the MMC. (d) The silicon cantilever containing the “V” shaped micro‐coil.

In the experiment, mice were anesthetized with isoflurane at a concentration of 4% for induction, followed by 0.9%–1.5% for maintenance. Their body temperature was maintained at 37°C throughout the experiment. After performing a craniotomy, a micro‐coil (Figure 2a) was inserted 200–250 µm vertically from the cortical surface using a micro manipulator. The coil was allowed to rest for minimum 30 min to stabilize within the tissue.

Both wide‐field imaging and two photon imaging confirmed no bleeding of tissue surrounding the insertion site, and single neurons were distinctly visible near the probe tip for subsequent analysis (Figure 8).

For stimulation, a micro‐coil connected to a function generator (Agilent 33250A) was used to apply a 1 kHz sinusoid wave, phase shifted by −90°, with a pulse duration of 1 ms. The stimulation frequency was fixed at 200 Hz across all mice recorded. The stimulation intensity we set to 150 mV, corresponding to 2–3 V output with a stimulation duration of 0.5∼0.6 ms. We performed 5–10 trials with inter trial interval 10 s.

### 2.4 TPM Imaging and Data Analysis

We used a two‐photon microscope (FluoView FVMPE‐RS, Olympus) with a femtosecond laser (Mai‐Tai DeepSee) and 25 × 1.05NA water‐immersion objective. We used wavelength between 850 and 920 nm, with image acquisition frequencies between 1.81 and 4.38 Hz. For the following analysis, we first performed motion correction, then manually segmented the cell bodies to extract the region of interest (ROI). We further defined a neuropil area that was 1.5 times larger than the center of the cell body ROI. This neuropil areas was subtracted from the original soma ROIs with a weight of 0.7 to robustly obtain neuronal response from somata. Significance was tested using one‐sample *t*‐test against zero to confirm negative response amplitude induced by stimulation.

## 3 Results

We initially used FEM to conduct thermodynamic, electromagnetic, and solid mechanics simulations of the MMC. Guided by these simulations, we fabricated tonearm‐like MMC, with the micro‐coil probe consisting of three main components: the cantilever, the arm, and the base (Figure 2). After assembling the MMC onto a PCB, it was implanted into the mouse brain for in vivo experiments.

### 3.1 Overall Design of the Tonearm‐Like Micro‐Coil

Main challenges of using TPM with magnetic micro‐coil neuromodulation for in vivo imaging are: (1) the micro‐coil must be aligned parallel with the vertically oriented pyramidal neurons (PNs) in layer 2/3 to maximize stimulation efficiency, (2) the micro‐coil must be adaptable to the small working distance by high‐resolution TPM imaging, (3) minimal invasiveness must be ensured to prevent significant tissue damage by the micro‐coil, and (4) the micro‐coil must not cast a large shadow and obscure TPM imaging, which requires minimizing the physical footprint of the coil to ensure clear visibility under the TPM.

To address these challenges, we implemented the following designs (Figure 2) based on the constraints from the experimental setup. (1) The cantilever and arm of the MMC are perpendicular to each other, forming a tonearm structure that ensures mechanical strength and allows for vertical insertion into the visual cortex; meanwhile, the V‐shaped coil design ensures stimulation intensity. (2) The cantilever has a length of 800 µm, ensuring compatibility with objective lenses with a 2 mm working distance while leaving a margin; the cantilever's width is 86 µm, and thickness is 70 µm, resulting in a very small cross‐sectional area. (3) We used biocompatible materials to minimize damage to brain tissue, and (4) shadows in the microscope's field of view were reduced by the small physical footprint of the coil.

### 3.2 Numerical Simulations for Device Design Feasibility and Guidance

Numerical simulations are crucial in the design and analysis of the micro‐coil devices used in neural stimulation. They provide detailed insights into the mechanical, electromagnetic, and thermal behaviors of the devices under experimental conditions. These simulations enable the prediction and optimization of device performance, ensuring safety and efficacy in biological environments. By assessing the distribution of electric fields, temperature changes, and mechanical stresses, the simulations guide design modifications that enhance stimulation efficiency, maintain structural integrity, and prevent thermal damage to surrounding tissues. To ensure reliable numerical modeling, mesh sensitivity analysis has been performed and presented in Supporting Information (Figure S1, S3 and S5).

Solid mechanics simulations investigate the mechanical stability and structural integrity of the tonearm‐like micro‐coil during insertion into brain tissue. Assessing stress distribution and displacement is critical to ensuring that the probe can withstand mechanical forces without failure, minimizing damage to the brain tissue. Stress and displacement analyses reveal the mechanical advantages of the tonearm‐like design. We compared our tonearm‐like probe to the Michigan design, which can be used to probe neural tissues deeper from the skin surface [67, 82, 83]. The dimensions of the tonearm device are given in Figure 2 and Table 1. An axial load at the tip is predefined as 10 mN following earlier studies to evaluate von Mises stress distribution and displacement magnitudes [84]. Linear buckling analysis was also performed to determine the critical loads for each design.

**FIGURE 3:**
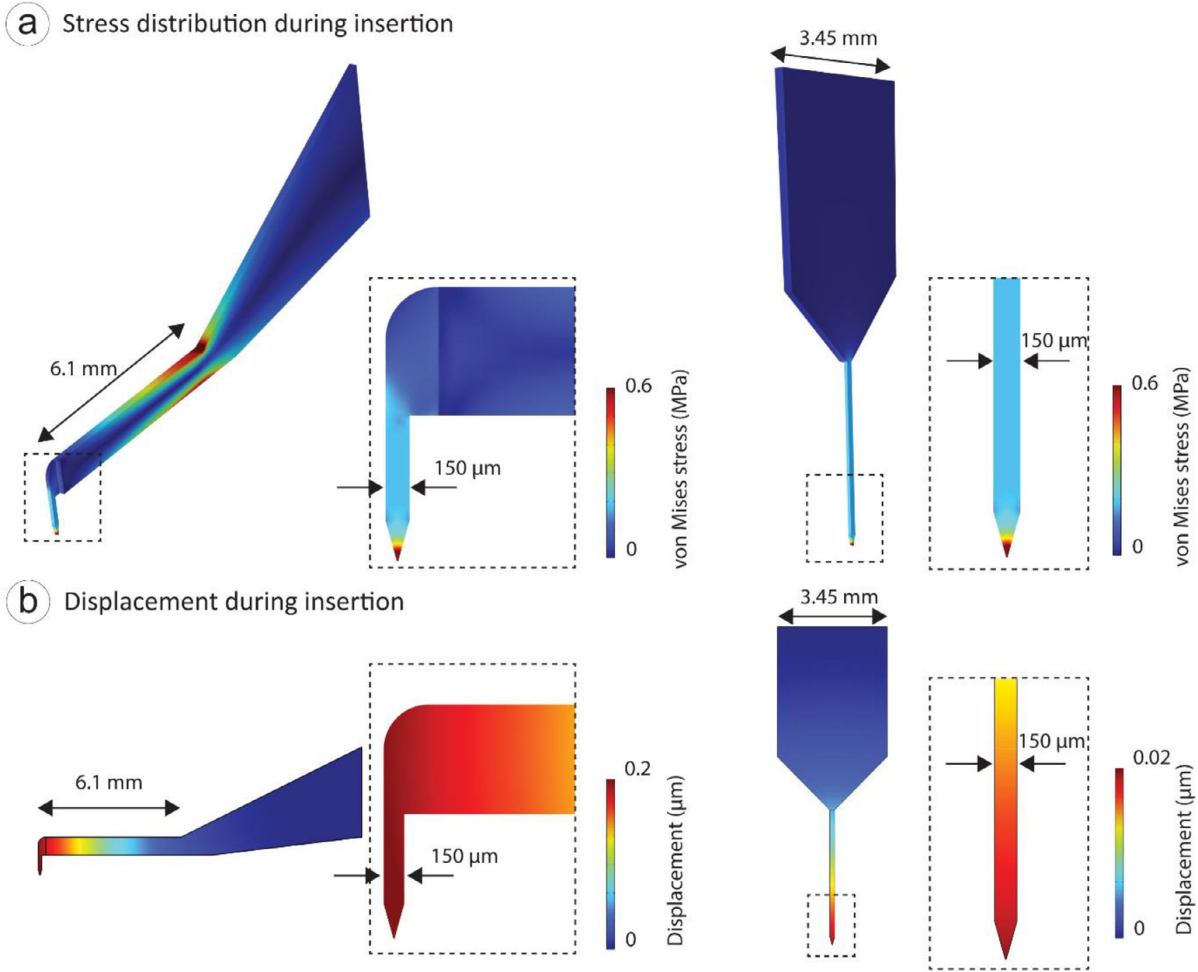
Mechanical consideration when comparing tonearm‐like probe with Michigan probe, showing distribution of (a) von Mises stress and (b) displacement magnitude with an axial load during insertion.

Figure 3a shows a more balanced von Mises stress distribution in the silicon base of the tonearm‐like probe compared to the Michigan design where stress is concentrated at the tip. Displacement analysis (Figure 3b) indicates that the tonearm‐like probe experiences slightly higher displacement under axial load, but linear buckling analysis confirms a 14‐fold higher critical load compared to the Michigan probe (Figure S2). Since the Michigan design are mechanically stable for most implantation experiments, our mechanical simulations suggested that the tonearm‐like probe would also be mechanically stable during implantation.

**FIGURE 4:**
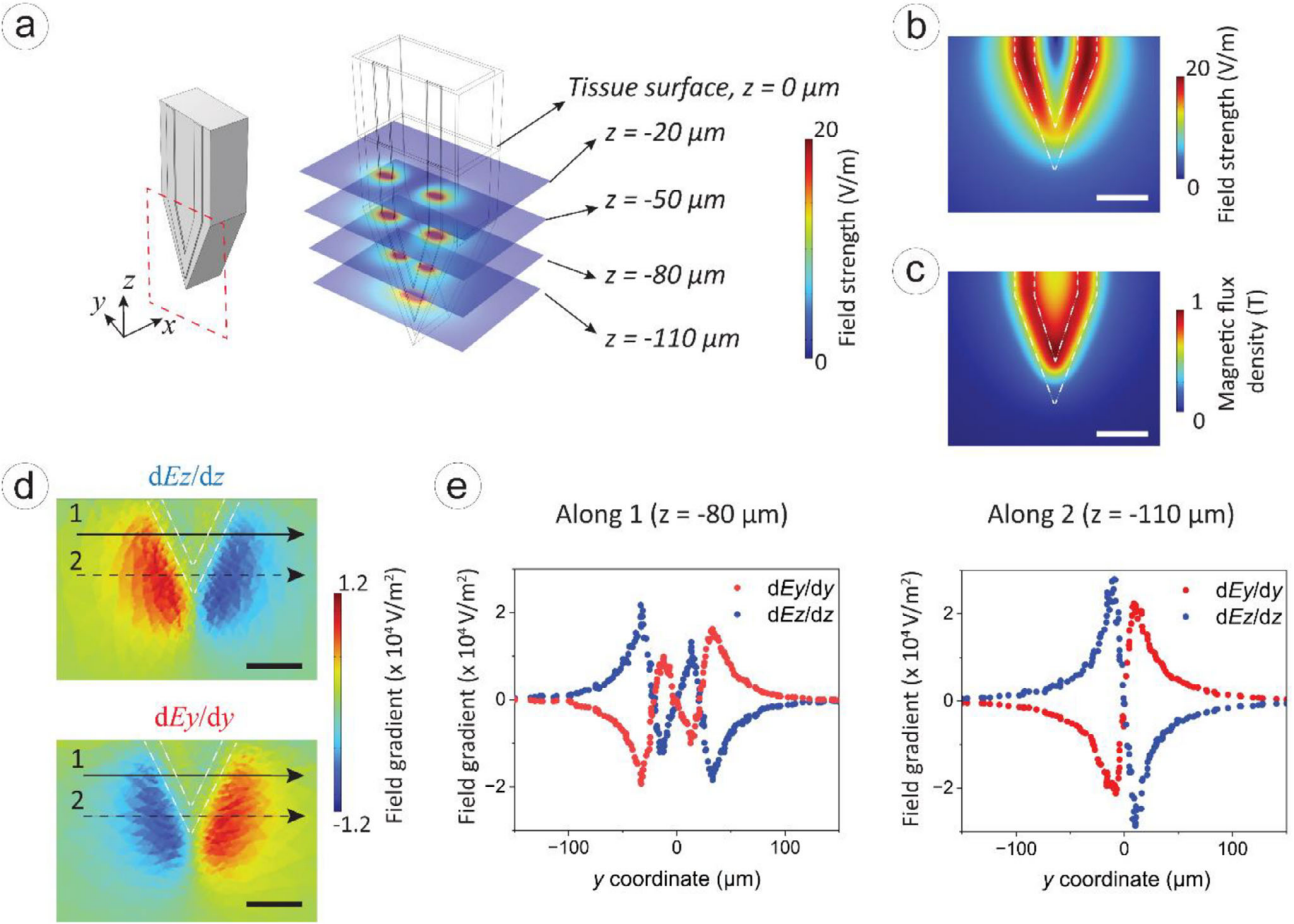
Electromagnetic simulations of proposed device. (a) An illustration of probe tip and simulated electric field distribution in different stimulation levels. (b) 2D distribution of electric field strength and (c) magnetic flux density in the red squared area illustrated in (a). (d) Electric field gradient dEz/dz and dEy/dy distribution in the red square area, where two levels 1 and 2 are illustrated. (e) 1D distribution of field gradient along lines 1 and 2.

Electromagnetic simulations were conducted to evaluate the electric field distribution generated by the micro‐coil. This is essential because efficient neuron stimulation requires a focused and sufficient electric field strength, especially around the probe tip. Understanding the electromagnetic characteristics helps optimize the coil design to ensure successful and targeted neuromodulation. The probe tip is immersed (140 µm deep) into a brain mimicking medium, which has an electrical conductivity of 0.276 S m^−1^ and a relative permittivity of 12 000 [85]. The aluminum wire and silicon substrate has electrical conductivities of 3.773 × 10^7^ S m^−1^ and 1 × 10^−^^1^ ^2^ S/m, respectively, while aluminum oxide and parylene C coatings are assumed to be non‐conductive. A time‐varying potential (peak: 120 mV, frequency: 200 Hz) was applied to one end of the aluminum wire. The electric field simulation results show the spatial distribution of the field generated by the V‐shaped micro‐coil at the probe tip. Figure 4a shows the electric field surrounding the metallic core, effectively stimulating the region near the tip surface. At stimulation depths of z = −20, −50, −80, and −110 µm, the electric field distribution remains concentrated along the metal traces, as shown in Figure 4b,c. Magnetic flux density is intensified at the tip region, correlating with the electric field patterns. Field gradient analysis in Figure 4d,e reveals peak field gradients of 2.0 × 10^4^ V m^−2^, surpassing the neuromodulation threshold of 1.1 × 10^4^ V m^−2^ [86, 87, 88].

**FIGURE 5:**
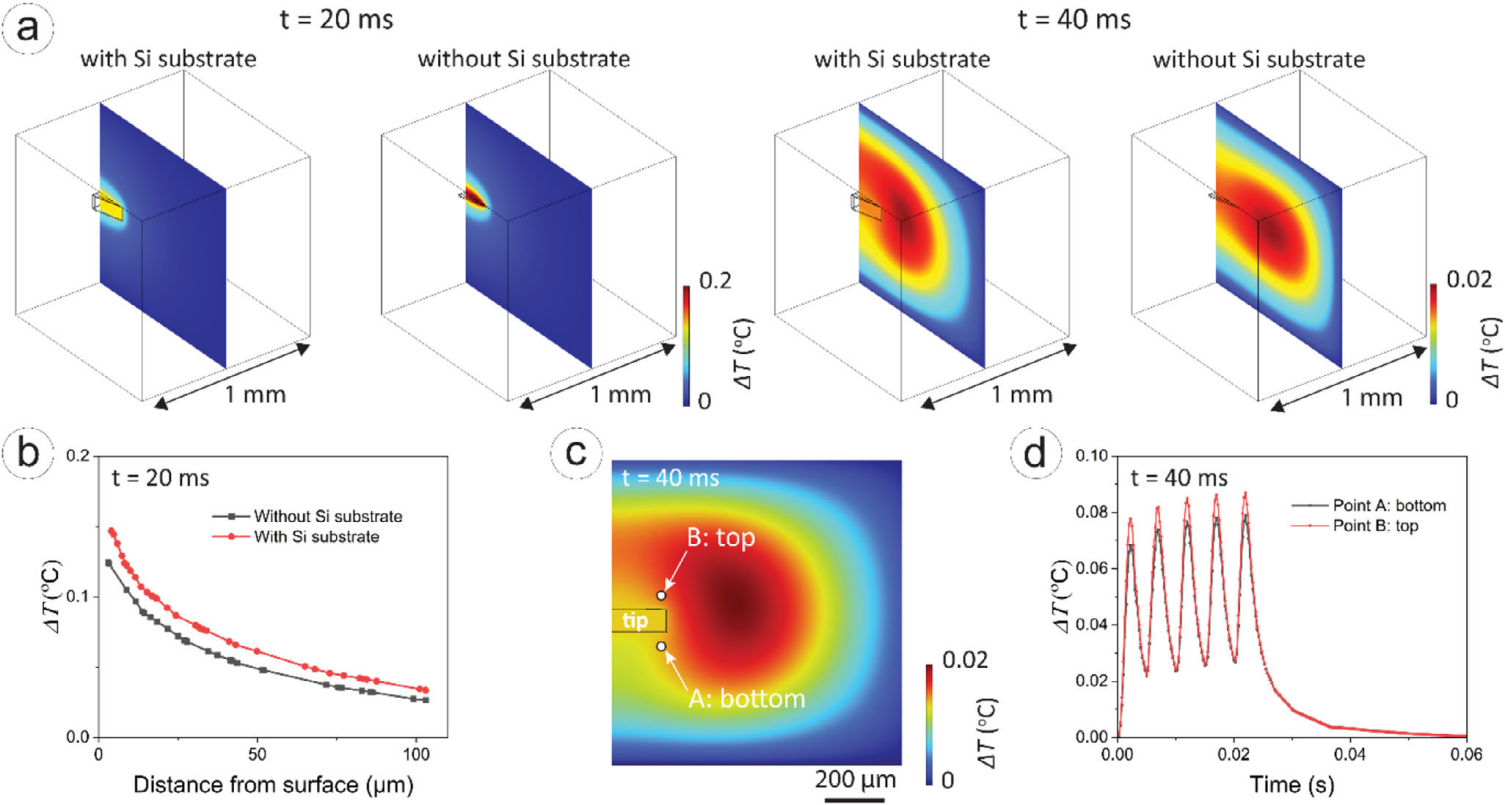
Temperature distribution simulated by FEM. (a) Temperature distribution at 1 and 40 ms. (b) Temperature map at the tip position at 1 ms. (c) Temperature evolution when a pulse train is applied for stimulation, two points A and B are compared, which are illustrated in (b).

Finally, heat transfer simulations were performed to assess the temperature changes induced by the micro‐coil during stimulation. High temperatures could damage surrounding brain tissue, making it crucial to quantify temperature fluctuations and ensure neuromodulation remains within safe limits. This simulation shows that our microcoils can function as thermal‐efficient devices, preventing excessive heat accumulation, as silicon substrates can act as a heat sink. The temperature distribution results, presented in Figure 5a, show a significant temperature increase localized around the tip surface during stimulation (t = 20 ms). With the silicon substrate, the maximum temperature increase was limited to 0.125°C, while the absence of the silicon substrate resulted in a 17% temperature increase. At t = 40 ms, after the pulse train, the temperature decreased due to the heat diffusion into surrounding tissue, and the post‐stimulation temperature was 0.02°C. Our results adhere to the ISO 14708‐1 standard for implantable devices, which limits the maximum allowable temperature rise to 2°C [89]. Material properties used in the simulations included thermal conductivities of 238 W m^−1^K (aluminum core), 130 W m^−1^K (silicon substrate), 35 W m^−1^K (aluminum oxide), 0.19 W m^−1^K (parylene C), and 0.45 W m^−1^K (brain tissue) [75]. A pulse train was applied to the micro‐coil (the waveform is presented in Figure S4), and the temperature evolution was monitored at two time points: during stimulation (t = 20 ms) and post‐stimulation (t = 40 ms). Comparisons were made between cases with and without the silicon substrate to evaluate its effectiveness as a heat sink.

### 3.3 MEMS Fabrication

**FIGURE 6:**
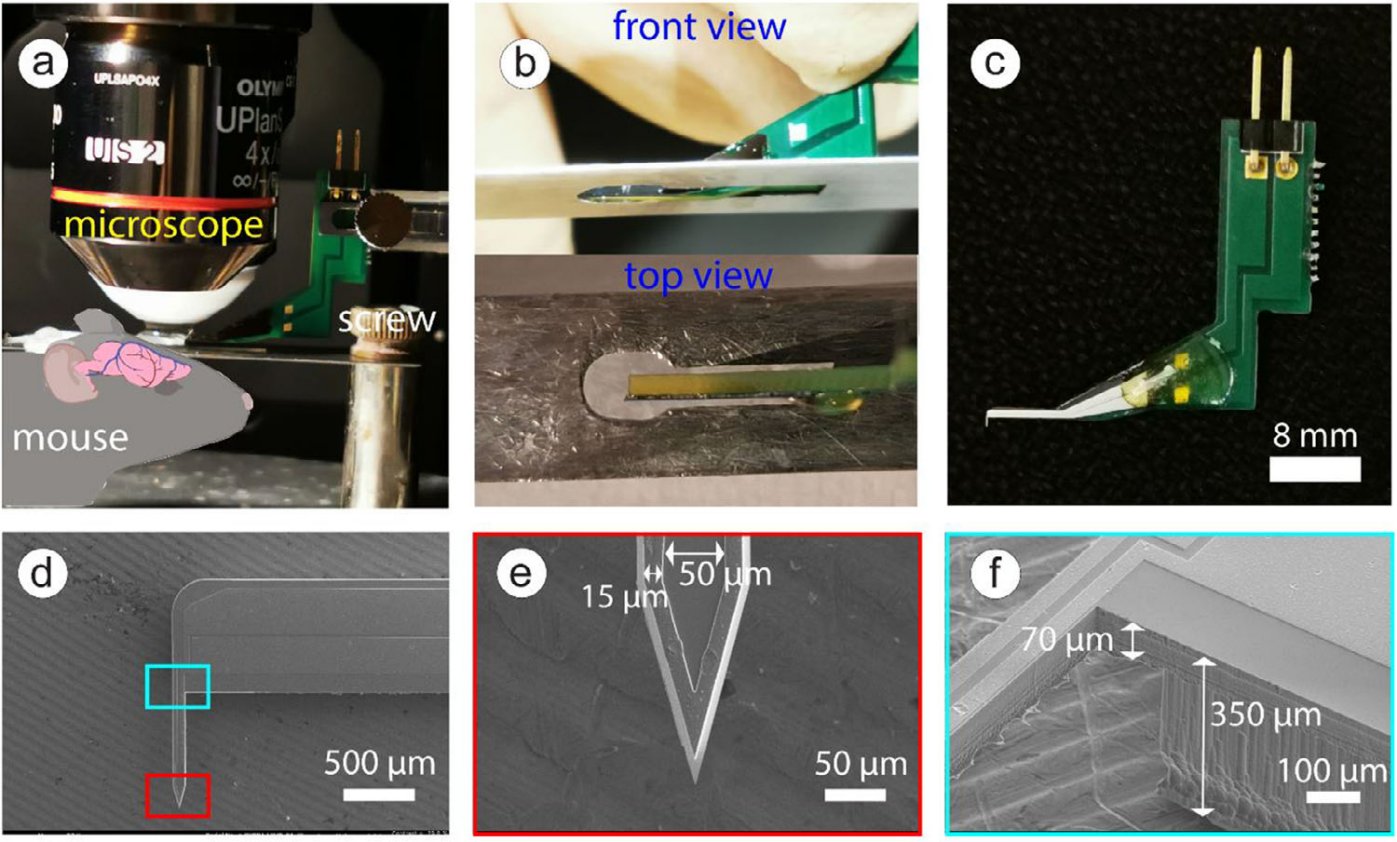
SEM and optical microscope images of the probe fabrication process. (a) Fabricated probe and experimental setup. Created in BioRender. Kim, K. (2025) https://BioRender.com/22lh3nz. (b) Custom‐designed headbar for probe adaptation. (c) Completed probe. Scale bar: 8 mm. (d) SEM image of the MMC, scale bar: 500 µm. (e) Enlarged view of the probe tip corresponding to the red box in d), showing an Al trace with a width of 15 µm. Scale bar: 50 µm. (f) The joint between the arm and cantilever, corresponding to the blue box in (d). The Si arm thickness is 70 µm, and the Si base thickness is 350 µm. Scale bar: 100 µm.

The MEMS probe was designed based on finite element modeling (FEM) analysis. Fabrication results of the key steps and the associated technological challenges are presented in Figure 6.

The fabricated MMC probe was configured according to Figure 6a for in vivo experimental implementation. Both the PCB and headbar were designed with complementary geometries (Figure 6b,c) to ensure precise mechanical interlocking. A V‐shaped half‐turn micro‐coil, comprising 15‐µm‐wide aluminum traces with 50‐µm intertrace spacing, was integrated at the cantilever tip. The cantilever thickness was precisely controlled at 70 µm through a multi‐step process of DRIE. This reduced cortical tissue damage during probe insertion. Concurrently, the support arm maintained a 350‐µm thickness to ensure structural rigidity.

To precisely control the margin between the Al trace and the edge of the Si cantilever (Figure 6e), ensuring it is small enough to minimize damage to brain tissue, we first used UV lithography to transfer the pattern onto an alumina layer. The excess alumina was then etched away to form a hard mask. At this stage, the Al trace was fully encapsulated between two layers of alumina. As we etched the Si to form the cantilever from the top, this process ensured that the margin between the Al trace and the Si cantilever edge could be accurately controlled to within 3 µm.

**FIGURE 7:**
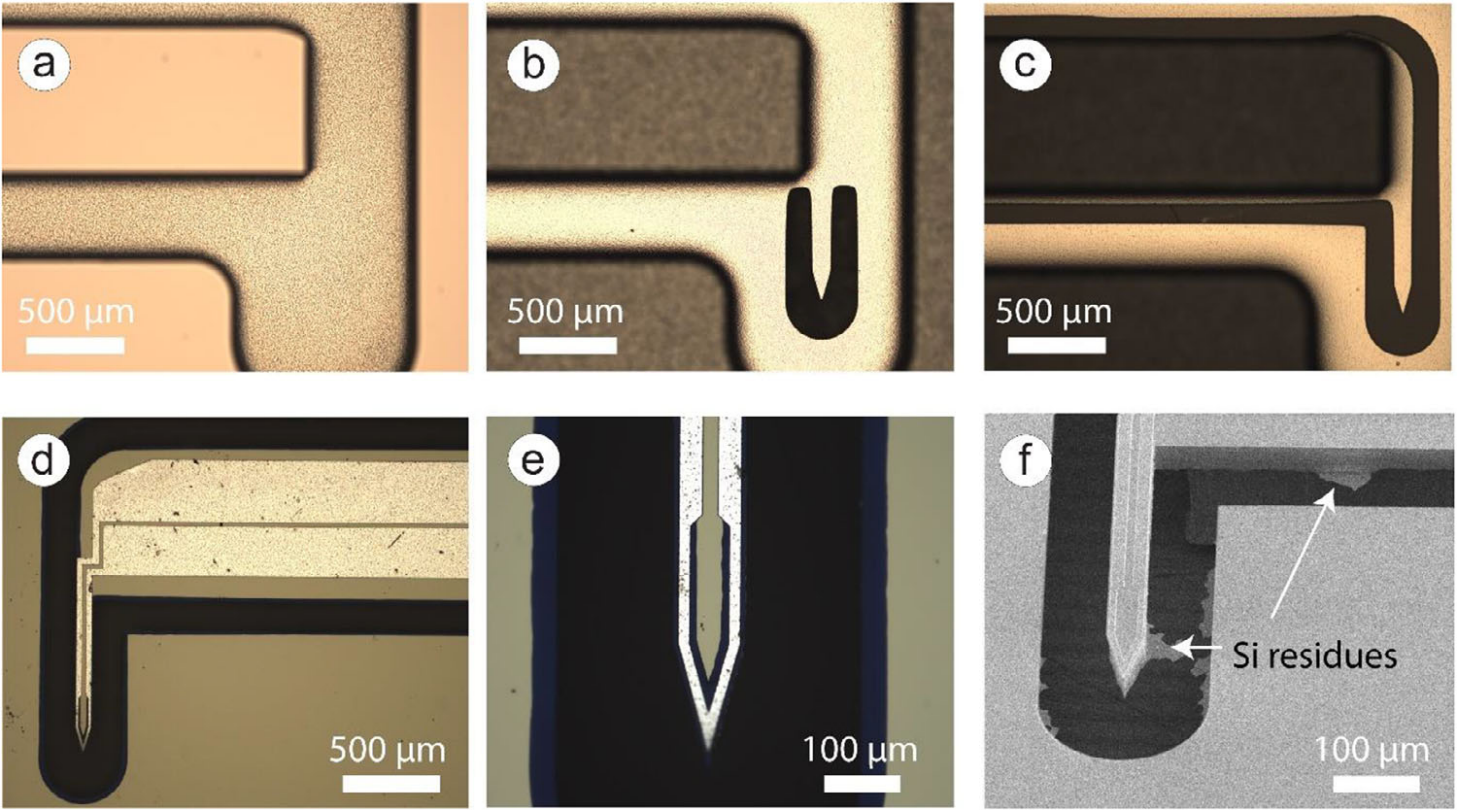
The silicon etching process: (a) Optical microscope images of the backside of the probe followingstep m. Scale bar: 500 µm. (b) Etch‐through was first observed around the silicon tip, during step n. Scale bar: 500 µm. (c)Remaining silicon was completely etched through, following step n. Scale bar: 500 µm. (d) Optical microscope image showing excessive etching of Si, captured from the top of the probe. Scale bar: 500 µm. (e) Enlarged view of the probe tip corresponding to the red box in (d), showing excessive etching of Si. Scale bar: 100 µm. (e) SEM image of Si residues. Scale bar: 100 µm. (f) SEM image of Si residues. Scale bar: 100 µm.

Employing a passivation treatment, sharp corners were replaced with arcs (Figure 7) to enhance roughness. This change specifically aimed to reduce the likelihood of wafer breakage occurring during the critical etching step m.

In step m, the thinnest feature of the design was measured at only 20 µm, hence an aluminum carrier was used to secure the wafer and allowed etching to continue on its backside. Altough the Al carrier prevented vacuum loss, which resulted in some degree of isotropic etching during the etch‐through process in the following step n, the Al trace, that was fully encapsulated in alumina, remained unaffected.

However, if step n did not completely etch through the Si, residual Si could remain, which would affect the probe's ability to penetrate brain tissue (Figure 7f). This issue needed to be carefully addressed to ensure the probe's functionality. Despite the challenges, this approach even allowed for further reduction in the cross‐sectional area of the Si cantilever.

Post‐fabrication, the MEMS micro‐coil was affixed to the PCB's bonding area using adhesive. Electrical continuity between the bonding pads on the micro‐coil and the PCB was then established with a gold ball wire bonding process. Epoxy was applied on the bonding wires for protection from potential damages Subsequentæy, the device was completely encapsulated with a 6‐µm‐thick layer of Parylene C. According to the simulation results, the DC resistance prior to wire bonding was found to be less than 2 Ω. The final resistance of the wire‐bonded assembly was measured to be 2 ± 0.5 Ω.

The electrical insulation testing was done in a phosphate‐buffered saline (PBS) solution, in which the resistance between a reference electrode and the microcoil was measured. The measured resistance exceeded the 210 GΩ capacity of the Keithley 6514 electrometer, indicating effective insulation. Following these tests, the MEMS probes were prepared for in vivo experiments.

### 3.4 Validation of Micro‐Coil Using In Vivo Two‐Photon Imaging in Mice

We further validated the feasibility of the tonearm micro‐coil using in vivo two‐photon imaging in the mouse visual cortex. It is important to note that our previous study characterized neuronal responsiveness [68], including cellular‐level response magnitude, dependence on stimulation intensity, and spatial patterns of inhibition. In contrast, the present work builds upon and extends those findings. Specifically, we evaluate the reliability and consistency of the stimulation‐induced suppression across multiple probes and repeated trials. Although no additional samples were collected, the results presented here emphasize the reproducibility of the suppressive effect across independent preparations.

**FIGURE 8:**
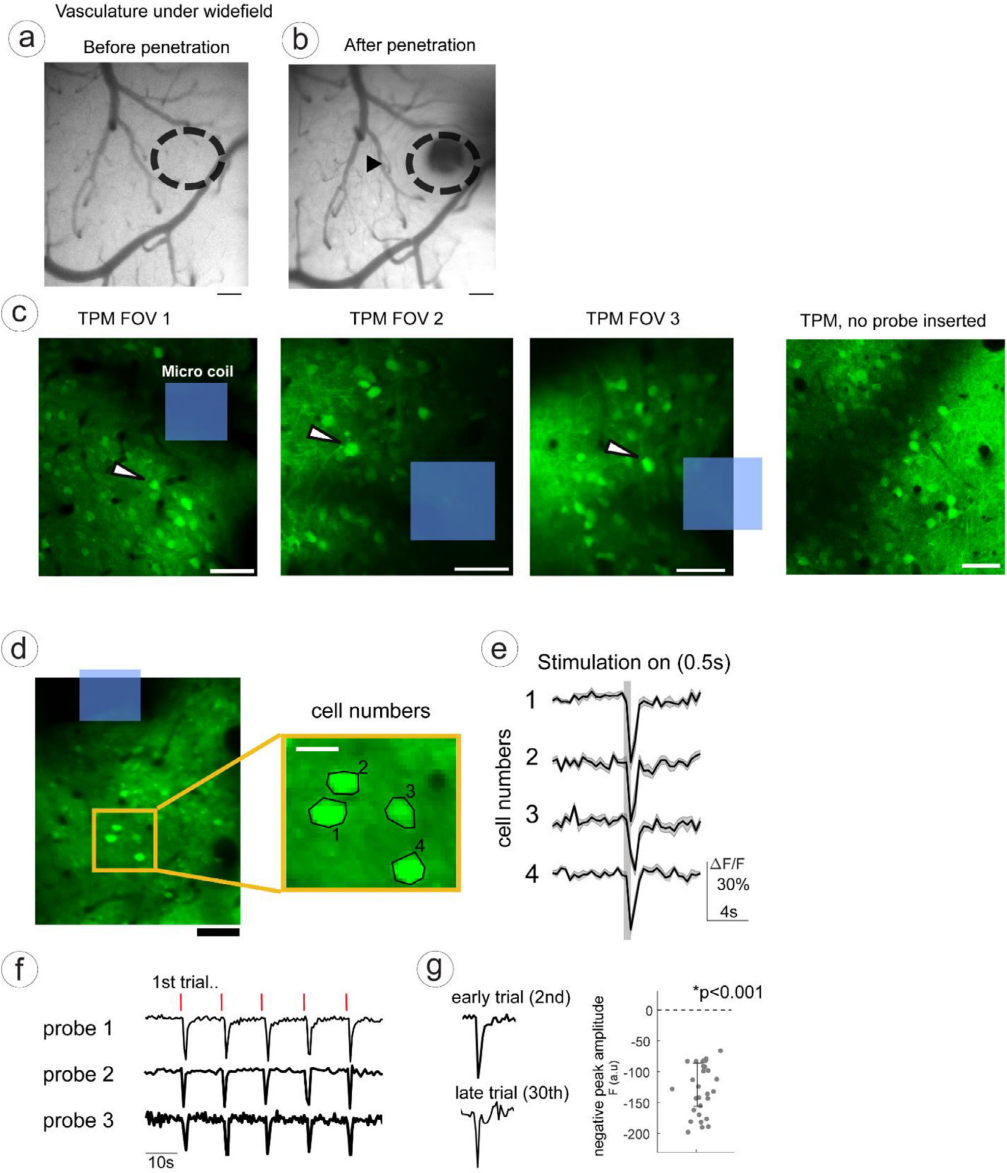
Validation of micro‐coil using in vivo two photon imaging in mice. (a,b) Vasculature under wide‐field imaging demonstrating low invasiveness and minimal bleeding. (a) Before probe insertion, dashed black circle indicates target area for microcoil penetration. (b) After microcoil penetration of up to 200 µm into the cortex. Scale bar: 50 µm. (c). Example two‐photon imaging fields. Typical two‐photon imaging fields of view from three different mice. Blue square indicates micro‐coil location. White arrow indicates a neuron (cell body). Scale bar, 50 µm. (d,e) Single cell showing robust suppression. (d). Individual neurons nearby the microcoil (d, yellow box) and corresponding response traces (e). Scale bar, 50 µm (d, left image) and 10 µm (d, right image). f) Response traces by three different probes (3 mice), red bar indicates stimulation timing. g) Response traces from early trial (second, upper trace) later trial (30th, lower trace). Right scatter plot shows total 30 trials and the negative peak amplitude (f, arbitrary unit) with significance (one dot equals one trial).

We highlight the successful vertical penetration with minimal invasiveness. The probe was small enough to be inserted between microvessels (50∼100 µm) in the densely vascularized mouse cortex (Figure 8a) without causing bleeding (Figure 8b). In both two‐photon and wide‐field imaging, even minor vessel damage can cause bleeding and darken the field of view, preventing further recordings.However, we did not observe such interference in our experiments. Moreover, the presence of the microcoil did not cast large shadows that could obstruct imaging, allowing us to clearly visualize individual neuronal cell bodies (Figure 8c,d) and extract their activity in response to stimulation.

During imaging, sinusoidal micro‐coil stimulation (1 kHz waveform, 200 Hz repetition rate, 150 mV input) was applied as described in the Methods. This stimulation reliably induced robust suppression of neural activity (Figure 8e), a response that was consistent across multiple probes and mice (Figure 8f, data from three probes intact in three different mice).

Aside from securing an imaging field of view with minimal invasiveness, our in vivo results further confirm that the stimulation‐induced suppression is highly robust and repeatable, persisting across up to 30 consecutive stimulation trials (Figure 8g, left). The recorded activity traces exhibited consistent negative deflections significantly below zero (one‐sample *t*‐test against zero, *p* < 0.001), demonstrating the reliability and reproducibility of the suppressive neuromodulatory effect elicited by the MMC probes.

## 4 Discussions

This study demonstrates the successful development and validation of a tonearm MMC system for precise cortical neuromodulation, addressing four critical challenges in integrated two‐photon microscopy applications. The optimized design features an 800‐µm cantilever with an 70 × 86 µm^2^ cross‐section, achieving compatibility with standard TPM objectives while maintaining mechanical robustness. Advanced MEMS fabrication achieved sub‐1 µm edge alignment precision through UV lithography patterning on alumina layers, where aluminum traces were fully encapsulated between dual alumina hard masks during silicon etching. This encapsulation strategy enabled precise control of the Al/Si cantilever interface margin (< 1 µm) while preventing isotropic etching effects, ultimately reducing the probe's cross‐sectional footprint to minimize tissue displacement.

Finite element method simulations guided critical design decisions. 1) Solid mechanics analysis revealed excellent stress distribution versus the Michigan probe, with a 14‐fold higher buckling resistance (critical load) despite comparable displacements under 50 mN axial force. 2) Electromagnetic modeling showed focused stimulation capability, generating peak electric field gradients of 2.0 × 10^4^ V/m^2^ at the probe tip, which is above the neuronal activation threshold. 3) Thermodynamic simulations ensured thermal safety, limiting temperature rise to 0.125°C, well below the ISO 14708‐1 2°C threshold.

The fabricated devices exhibited exceptional electrical performance (2 ± 0.5 Ω resistance) and insulation integrity (>210 GΩ in saline solution), attributable to the alumina‐aluminum encapsulation architecture and final parylene‐C coating. In vivo validation across 217 visual cortex neurons in mice revealed significant neuromodulatory effects. The vertically inserted probe maintained optical accessibility, with <5% shadow area in TPM fields. These results collectively establish the MMC as a neuromodulation tool that synergizes electromagnetic stimulation precision with TPM‐compatible form factors, overcoming traditional trade‐offs between stimulation efficiency and imaging compatibility.

In additional to added functionalities, this work demonstrates critical improvements in three domains (Table 3): (1) Balanced electrical performance—Achieved 2 Ω DC resistance, while maintaining >210 GΩ insulation. (2) Safer microfabrication—Eliminated toxic buffered HF (BHF) via dry etching protocols. (3) Precision scaling—decreasing the maximal distance between the Al trace to the cells to 7 µm using maskless lithography, enabling low‐invasive 86 × 70 µm^2^ implant cross‐sections without sacrificing mechanical stability.

**TABLE 3:** MEMS micro‐coil fabrication technology comparison.

	Lee et al., (2019) [87]	Sugai et al., (2020) [90]	Liu et al., (2023) [67]	This work
Si wafer substrate	50‐µm‐thin	Silicon‐ on‐oxide wafer	Double‐side polished	Double‐side polished
lithography	Photomask	Photomask	Maskless	Maskless
coil wire	2‐µm‐thick Au	200‐nm‐thick Pt	800‐nm‐thick Al	1800‐nm‐thick Al
DC resistance	50 Ω	1 kΩ	4 Ω	2 Ω
leak resistance	200 MΩ	2 MΩ	>210 GΩ	>210 GΩ
BHF etch	No	Yes	Yes	No
Probe cross‐section	50 × 100 µm^2^	50 × 100 µm^2^	60 × 320 µm^2^	70 × 86 µm^2^
Max coil to tissue distance	5 µm	5 µm	56 µm	7 µm

Our MMC‐based technology complements existing INM approaches [42, 43, 44, 91] by offering distinct operational advantages in thermal‐sensitive scenarios. While INM excels in deep‐tissue penetration through infrared transparency, our system provides significant lower thermal perturbation (0.125°C vs. INM's 1°C–2°C) critical for longitudinal cortical studies requiring repeated stimulation under prolonged two‐photon imaging. The electromagnetic actuation mechanism eliminates INM‐associated photobleaching risks [44], enabling artifact‐free calcium imaging during suppression protocols. Conversely, INM remains preferable for subcortical targets beyond the MMC's current 800 µm reach. Together, these modalities enable layered experimental designs: magnetic neuromodulation for cortical circuit interrogation with simultaneous TPM readouts, and INM for deeper nuclei modulation, synergistically mapping multi‐scale neural dynamics.

## 5 Conclusion

In summary, the advanced micro‐coil implant presents a viable and highly effective solution for targeted neuronal inhibition with significant implications for both therapeutic interventions and neuroimaging. By overcoming the limitations of prior technologies, it provides a pathway for broader application in neuroengineering. While the current study offers comprehensive insights and answers to our research questions, future work will focus on expanding the scope of this technology, exploring diverse in vivo applications, and addressing new questions that arise as this field continues to evolve. (Supporting Information)

## Author Contributions

X.L.† fabricated the micro‐coil, performed experiments, and wrote the manuscript; B.C.† conducted numerical simulations, wrote the manuscript, and acquired funding; K.K.† performed experiments, analyzed data, and wrote the manuscript; G.A.E.A. contributed to manuscript writing; S.G. fabricated the micro‐coil; C.C. and A.H. conceived the study, supervised the work, wrote the manuscript, and acquired funding; all authors reviewed and edited the original draft.

## Funding

This study was supported by the Lundbeck Foundation (R345‐2020‐1440, R402‐2022‐1530, and R305‐2023‐1125), Danish National Research Foundation (1133‐00016B), the Novo Nordisk Foundation (NNF24OC0092323, NNF20OC0064289).

## Ethical Statements

We used laboratory animals and state that the animal's care was in accordance with 473 institutional guidelines.

## Conflicts of Interest

The authors declare no conflicts of interest.

## Supporting information




**Supporting File**: smll72864‐sup‐0001‐SuppMat.docx.

## Data Availability

The data that support the findings of this study are available from the corresponding author upon reasonable request.
