# Supplementary material for: “Tone‐Arm” Neuromodulation MEMS Microcoil for In Vivo Imaging
Source: Small. 2026 Feb 15;22(22):e14159. doi: 10.1002/smll.202514159 (PMC13089094; doi:10.1002/smll.202514159)
Supplement: Supplementary file 1 — Supporting File: smll72864‐sup‐0001‐SuppMat.docx. [file SMLL-22-e14159-s001.docx]

Supplementary materials

**“Tone-arm” Neuromodulation MEMS Microcoil for in-vivo imaging**

**Numerical simulation**

1. *Solid mechanics simulation*

Different simulation methods have been reported before to investigate the insertion process of a neural implant inside the brain tissues, where different adhesion properties (Subbaroyan et al., 2005), hyperelastic properties of brain tissues (Hamzavi et al., 2013), and friction conditions at probe-tissue interfaces (Singh et al., 2016) were used for more realistic modeling. Instead of investigating the implantation dynamics, here we focus on the mechanical properties of the microcoil probe by applying a load at the tip area, which is a widely used method to optimize probe designs (Draz et al., 2018; Nabaei et al., 2020).

For solid mechanics simulations, we are using a 3D linear elastic model with a predefined isotropic Young’s modulus of 170 GPa for silicon. The governing equations are based on a stationary Lagrangian version of Newton’s second law of motion, which is written as:

$$\nabla\cdot\left( FS \right)^{T}+\boldsymbol{F}_{\boldsymbol{V}}=0$$

$$\boldsymbol{F}=\boldsymbol{I}+\nabla\mathbf{u}$$

Here *F* is the deformation gradient, *S* is the stress tensor, *F_V_* is the body force, *u* is the displacement field, and ***I*** is an identity tensor. For buckling analysis, we used linearized buckling analysis by evaluating the first eigenmodes under the applied load and then compare the critical load factor.

The meshed “tone-arm” probe is presented in Fig. S1, and the geometry is based on the fabricated device. The tip of the probe is refined in mesh sizes for higher simulation accuracy. We used fixed boundary conditions at the end of the device to mimic the real implantation process. A compressive boundary load of 10 mN is applied in the z-direction at the end of the tip (tip radius of 6 µm, thickness of 70 µm). We used a total of 13357 elements and 3663 vertices for meshing tone-arm devices, and we performed a mesh sensitivity analysis to evaluate maximum von Mises stress with different element numbers; we can see that the result is relatively stable when the number of elements is above ~ 6000.


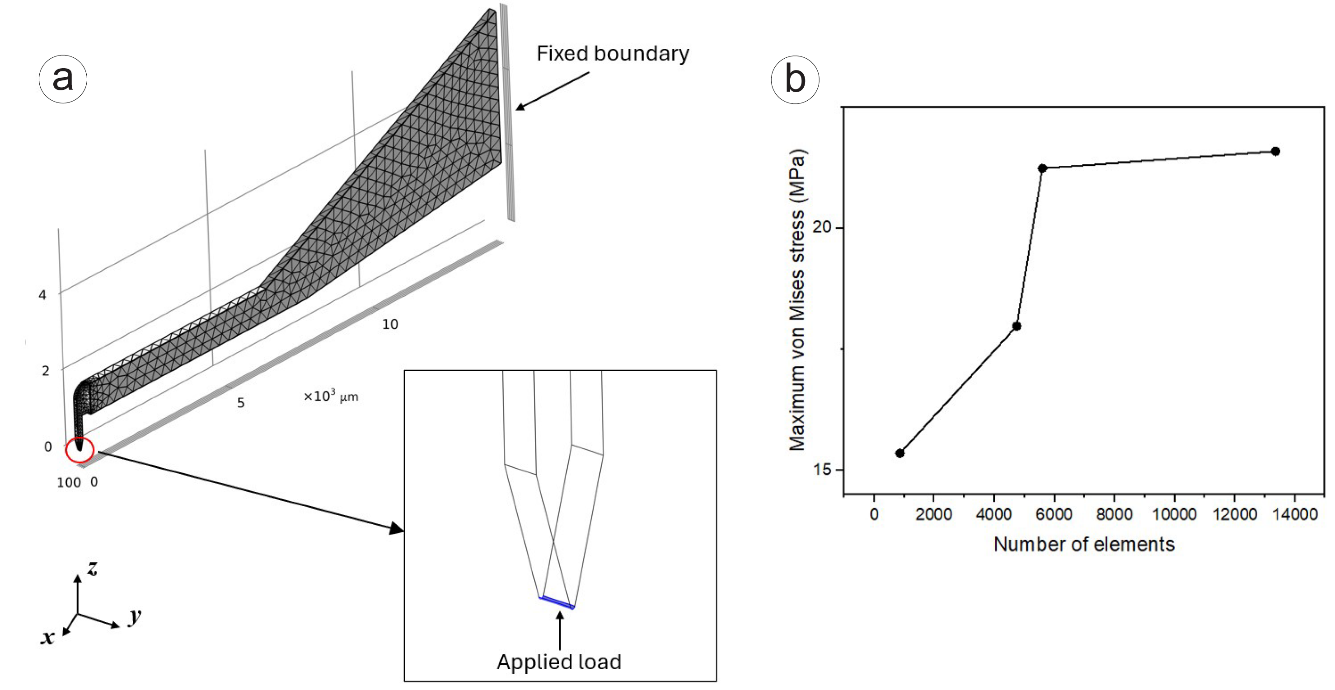


**Fig. S1.** (a) Meshed “tone-arm” microcoil implant, a zoom-in image showing the applied point load at the probe tip. (b) Mesh sensitivity analysis regarding maximum von Mises stress inside the device.

With the same boundary conditions, we performed a linearized buckling analysis and evaluated the critical load factor (CLF) for “tone-arm” and straight probe designs. The tip deformation is presented in Fig. S2, and a higher CLF can be achieved with “tone-arm” design, which is 13.97 times higher than the straight probe design, implying a much higher mechanical stability when a load is applied at the tip.


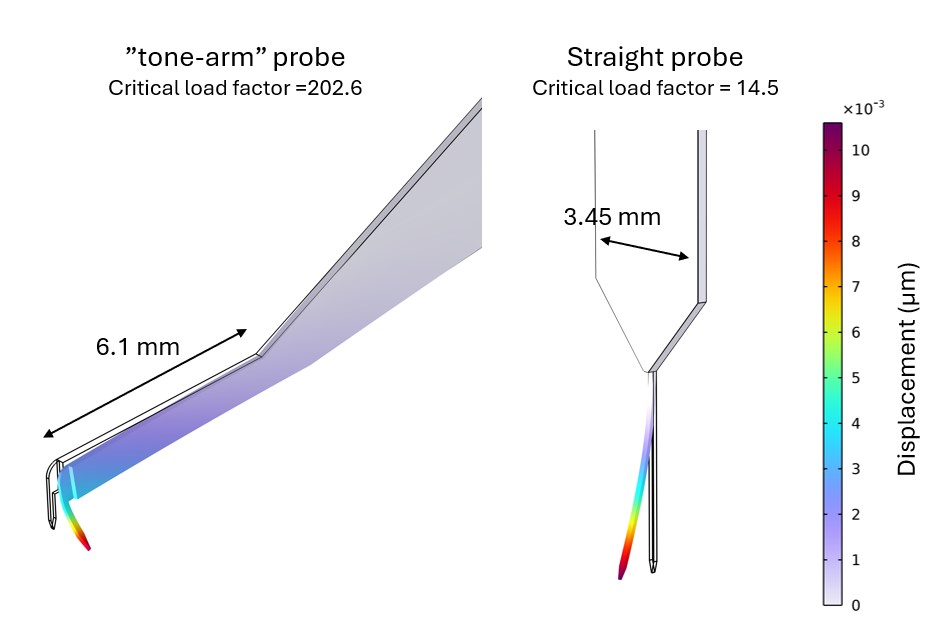


**Fig. S2.** Linear buckling analysis comparing “tone-arm” probe and straight probe. The displacement is displayed (a scaling factor of 80000 is used to highlight the tip deformation).

1. *Electromagnetic simulation*

For 3D electromagnetic simulations of the microcoil device, we used the magnetic and electric fields module in COMSOL and studied in a frequency domain. The governing equations are Maxwell equations as

$$\nabla\times\boldsymbol{H}=\boldsymbol{J}$$

$$\boldsymbol{B}=\nabla\times\boldsymbol{A}$$

$$\boldsymbol{J}=\sigma\boldsymbol{E}$$

$$\nabla\cdot\boldsymbol{J}=0$$

We used a voltage excitation for the coil analysis, and the current density is given as

$$J=\frac{N(V_{coil}+V_{ind})}{AR_{coil}}$$

Where *N* is the number of turns specified, *V_coil_* is the input voltage for coil excitation, and we used 120 mV. *V_ind_* is the induced voltage in the coil, *A* is the cross-section area, and *R_coil_* is the resistance of the coil. The geometry of the microcoil is based on the actual device as fabricated, and the tip is surrounded by a cylinder-like domain with a diameter of 1 mm and depth of 1 mm. The top plane of the cylinder was set with insulating conditions, and a virtual domain was set for the rest of the cylinder domain, with a stretching factor of 10 to account for the size of a mouse brain. The cross-section area of the microcoil device was set with a symmetry boundary condition. We used 610483 elements with 103993 vertices to perform the simulation, and the geometry is presented in Fig. S3 together with generated meshes. A mesh sensitivity analysis was also performed, and the maximum electric field strength on yz-plane is evaluated (2 µm away from the probe surface). It can be seen that the result converges when more than 600000 elements are generated for meshing.


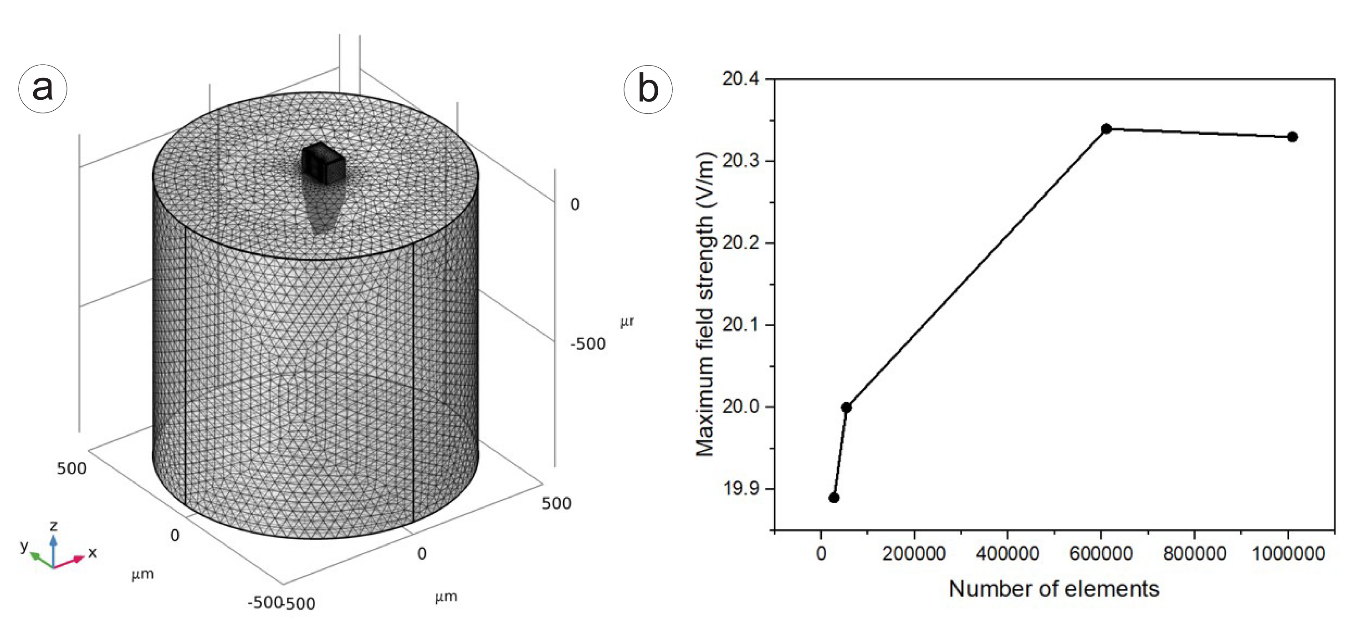


**Fig. S3.** (a) Meshed model for electromagnetic simulations of microprobe inside a cylinder region of brain tissue. (b) Mesh sensitivity analysis regarding maximum field strength in yz-plane, with a distance of 2 µm away from probe surface.

1. *Heat transfer simulation*

For heat transfer simulations with microcoil devices in a time domain, we used the multiphysics electromagnetic heating module, where an AC/DC electric current module is coupled with a heat transfer in solid module. The resistive heating was considered, and the governing equation is given as:

$$\rho C_{P}\frac{\partial T}{\partial t}+\rho C_{P}\boldsymbol{u}\cdot\nabla T=\nabla\cdot\left( k\cdot\nabla T \right)+Q_{e}$$

Here ρ is the density of the medium, *C_p_* is the specific heat capacity, *T* is the medium temperature, **u** is the velocity vector of translational motion, *k* is the thermal conductivity, and *Q_e_* is the amount of thermal energy generated by a current density of ***J***, and $Q_{e}=\boldsymbol{J}\cdot\boldsymbol{E}$, where E is the local electric field. The electric field simulation is based on following governing equations:

$$\nabla\cdot\boldsymbol{J}=Q$$

$$\boldsymbol{J}=\sigma\boldsymbol{E}+\frac{\varepsilon_{0}\varepsilon\partial\boldsymbol{E}}{\partial t}+\boldsymbol{J}_{\boldsymbol{e}}$$

Where *J_e_* is the externally generated current density, and *Q* specifies the current source.

Our simulation is based on conductive heat transfer without considering convection (**u** = 0). The geometry and dimensions are based on the microcoil device used in experiments, and the tip of coil device is surrounded by a cube-like domain with size of 1 mm × 1 mm × 1 mm. For electric source input, we performed a pulse train of 5 pulses with peak potential of 120 mV, the pulse duration was 1 ms and period 5 ms (as shown in Fig. S4). All the materials are set with an initial temperature of 25 ˚C to mimic the body temperature of mice.


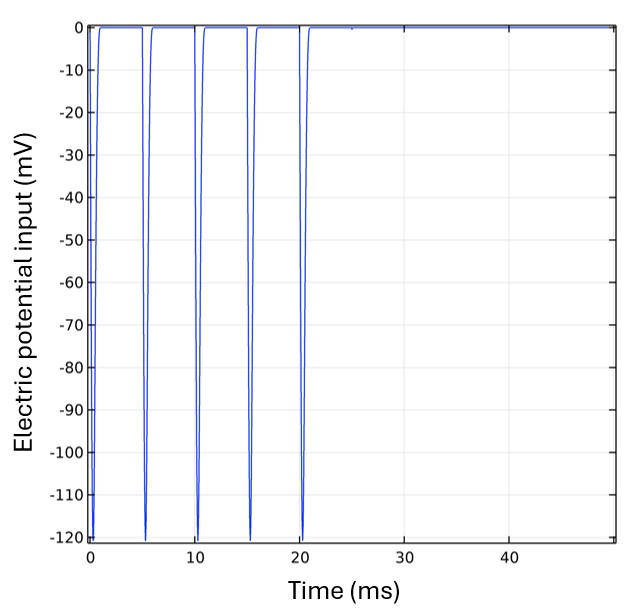


**Fig. S4.** input electric signal for time domain resistive heating simulations

A symmetry boundary condition is set for the xy-plane as shown in Fig. S5 (z-direction corresponds to the insertion axis of the microcoil device), where the generated meshes in microcoil-brain interfaces are also presented. The symmetry boundary is preset to consider the length of the real implant device, and the rest of the boundary planes of the cube-like domain are set with virtual domain conditions, with a stretching factor of 10 to account for the brain size of a mouse. A total number of 237355 elements are used for meshing with 41556 vertices. A mesh sensitivity analysis is performed regarding the maximum temperature increase during the pulse train, and it can be seen that the results converge when the number of element is beyond 200000.


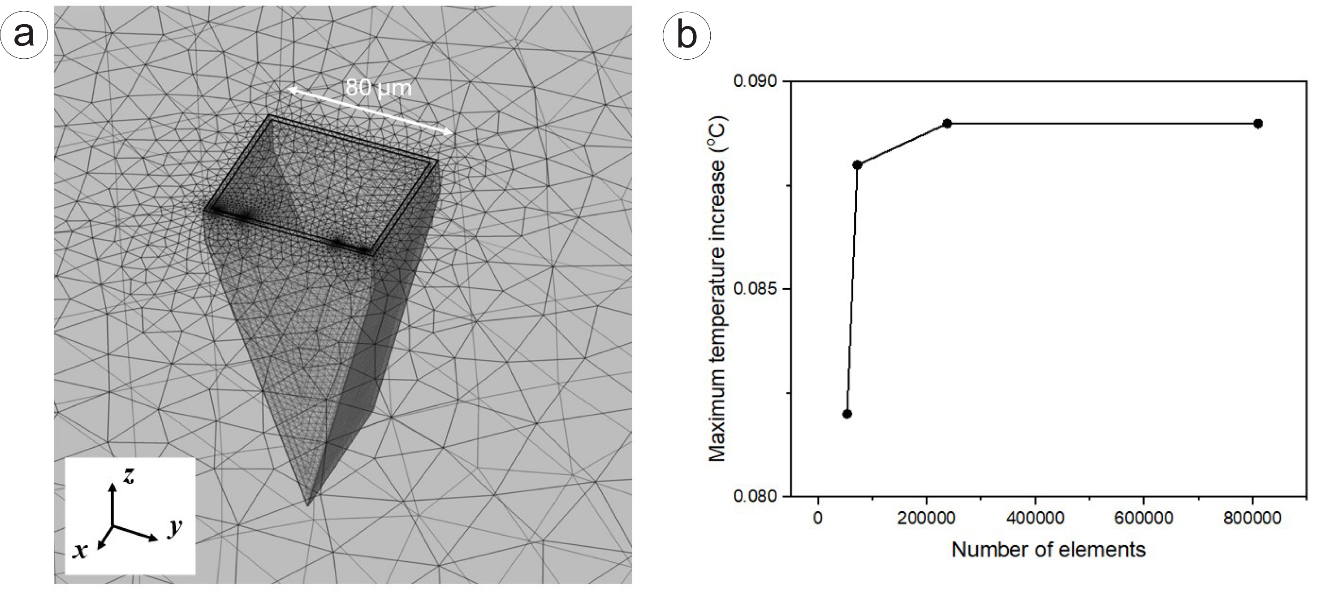


**Fig. S5.** (a) Meshes generated at the microcoil-brain interface. (b) Mesh sensitive analysis regarding maximum temperature increase.

**References**

Draz, H.H., Gabran, S.R.I., Basha, M., Mostafa, H., Abu-Elyazeed, M.F., Zaki, A., 2018. Comparative mechanical analysis of deep brain stimulation electrodes. Biomed Eng Online. https://doi.org/10.1186/s12938-018-0557-6

Hamzavi, N., Tsang, W.M., Shim, V.P.W., 2013. Nonlinear elastic brain tissue model for neural probe-tissue mechanical interaction, in: International IEEE/EMBS Conference on Neural Engineering, NER. https://doi.org/10.1109/NER.2013.6696134

Nabaei, V., Panuccio, G., Heidari, H., 2020. Neural microprobe device modelling for implant micromotions failure mitigation, in: Proceedings - IEEE International Symposium on Circuits and Systems. https://doi.org/10.1109/iscas45731.2020.9180497

Singh, S., Lo, M.C., Damodaran, V.B., Kaplan, H.M., Kohn, J., Zahn, J.D., Shreiber, D.I., 2016. Modeling the insertion mechanics of flexible neural probes coated with sacrificial polymers for optimizing probe design. Sensors (Switzerland) 16. https://doi.org/10.3390/s16030330

Subbaroyan, J., Martin, D.C., Kipke, D.R., 2005. A finite-element model of the mechanical effects of implantable microelectrodes in the cerebral cortex. J Neural Eng 2. https://doi.org/10.1088/1741-2560/2/4/006
